# Genome Engineering Technology for Durable Disease Resistance: Recent Progress and Future Outlooks for Sustainable Agriculture

**DOI:** 10.3389/fpls.2022.860281

**Published:** 2022-03-17

**Authors:** Qurban Ali, Chenjie Yu, Amjad Hussain, Mohsin Ali, Sunny Ahmar, Muhammad Aamir Sohail, Muhammad Riaz, Muhammad Furqan Ashraf, Dyaaaldin Abdalmegeed, Xiukang Wang, Muhammad Imran, Hakim Manghwar, Lei Zhou

**Affiliations:** ^1^State Key Laboratory for Managing Biotic and Chemical Threats to the Quality and Safety of Agro-products, Institute of Agro-product Safety and Nutrition, Zhejiang Academy of Agricultural Sciences, Hangzhou, China; ^2^Key Laboratory of Monitoring and Management of Crop Disease and Pest Insects, College of Plant Protection, Ministry of Education, Nanjing Agricultural University, Nanjing, China; ^3^College of Plant Science and Technology, Huazhong Agricultural University, Wuhan, China; ^4^Institute of Biology, Biotechnology, and Environmental Protection, Faculty of Natural Sciences, University of Silesia, Katowice, Poland; ^5^State Key Laboratory for Conservation and Utilization of Subtropical Agro-bioresources, Root Biology Center, College of Natural Resources and Environment, South China Agricultural University, Guangzhou, China; ^6^State Key Laboratory of Subtropical Silviculture, Zhejiang A&F University, Hangzhou, China; ^7^Department of Botany and Microbiology, Faculty of Science, Tanta University, Tanta, Egypt; ^8^College of Life Sciences, Yan’an University, Yan’an, China; ^9^Key Laboratory for Conservation and Utilization of Subtropical Agro-Bioresources, College of Agriculture, South China Agriculture University, Guangzhou, China; ^10^Lushan Botanical Garden, Chinese Academy of Sciences, Jiujiang, China

**Keywords:** plant pathogen, genome editing, CRISPR-Cas system, pesticide, disease resistance

## Abstract

Crop production worldwide is under pressure from multiple factors, including reductions in available arable land and sources of water, along with the emergence of new pathogens and development of resistance in pre-existing pathogens. In addition, the ever-growing world population has increased the demand for food, which is predicted to increase by more than 100% by 2050. To meet these needs, different techniques have been deployed to produce new cultivars with novel heritable mutations. Although traditional breeding continues to play a vital role in crop improvement, it typically involves long and laborious artificial planting over multiple generations. Recently, the application of innovative genome engineering techniques, particularly CRISPR-Cas9-based systems, has opened up new avenues that offer the prospects of sustainable farming in the modern agricultural industry. In addition, the emergence of novel editing systems has enabled the development of transgene-free non-genetically modified plants, which represent a suitable option for improving desired traits in a range of crop plants. To date, a number of disease-resistant crops have been produced using gene-editing tools, which can make a significant contribution to overcoming disease-related problems. Not only does this directly minimize yield losses but also reduces the reliance on pesticide application, thereby enhancing crop productivity that can meet the globally increasing demand for food. In this review, we describe recent progress in genome engineering techniques, particularly CRISPR-Cas9 systems, in development of disease-resistant crop plants. In addition, we describe the role of CRISPR-Cas9-mediated genome editing in sustainable agriculture.

## Introduction

Phytopathogens are one of the most common causes of plant diseases and pose a threat to global agricultural prosperity, as well as the safety of agro-based products. Plant diseases, caused by phytopathogenic bacteria, fungi, nematodes, viruses, invertebrate pests, and weeds account for approximately 20–40% of losses in agricultural crop yields worldwide (Ali et al., 2021, 2022). In the past few years, advances in crop breeding have provided a number of new technologies in the food and agriculture industry. Crops not only provide food for human consumption but also provide fuel and animal feed. The world population is expected to reach 9.6 billion by 2050, with a rise in global food demand by 100 to 110% compared with that in 2005 (Savary et al., 2019). However, current reductions in the extent of cultivable arable land and increasing water deficiencies highlight the urgency for innovate genome editing technologies in crop breeding for sustainable agriculture production. Moreover, given the emergence of new pathogens and development of resistance in existing pathogens, plant breeders, pathologists, and horticulturists need to develop different approaches to produce new cultivars with novel heritable mutations. Although traditional breeding practices have for long played a vital role in crop improvement, these typically involve prolonged laborious artificial planting over multiple generations.

Compared with conventional breeding, genetic engineering, which entails the use of biotechnology for direct editing of the genetic material of organisms (Christou, 2013), has numerous benefits. First, it can facilitate the insertion, deletion, modification, disruption, or fine-tuning of particular genes of interest and causes minimal, if any, undesirable alterations in the remaining crop genome (Hussain et al., 2021). Moreover, crops with desired traits can be obtained within fewer generations. Second, genetic engineering requires the exchange of genetic material between species. Consequently, the initial genetic material that can be used in this phase is not restricted to a single organism (Dong and Ronald, 2019). Third, in the process of genetic modification, plant transformation can introduce new genes into vegetatively propagated crops, including cassava (*Manihot esculenta*), potato (*Solanum tuberosum*), and banana (*Musa* sp.). Collectively, genetic engineering, thus, represents a potentially effective approach for enhancing the resistance to plant pathogens (Dong and Ronald, 2019).

Numerous aspects of crop genetic engineering are dependent on either traditional transgenic techniques or new genome-editing technologies. Using traditional transgenic methods, genes encoding proteins associated with required agronomic characteristics are inserted into random positions within the genome *via* transformation processes (Lorence and Verpoorte, 2004). These methods typically generate variants containing foreign DNA. In contrast, genome editing facilitates the modification of endogenous plant DNA at specific targets *via* deletion/insertion and replacement of the requisite DNA fragments (Barrangou and Doudna, 2016). In certain parts of the world, including the United States (USDA, 2018), Argentina (Agriculture, Livestock, Fisheries and Food Secretariat, Argentina, 2015), and Brazil, genome-edited plants that do not contain foreign DNA are exempt from regulatory measures applicable to genetically modified plants (CTNBio, 2018) and, accordingly, have a status equivalent to that of crop plants developed using traditional breeding techniques (Orozco, 2018). Despite these differences in regulatory practices, however, both traditional transgenic and new genome editing strategies represent important crop enhancement methods.

During the course of evolution, plants have developed multi-layer protective mechanisms against microbial pathogens (Chisholm et al., 2006; Jones and Dangl, 2006). For example, pre-formed physiological barriers and their enhancement prevent possible pathogens from entering the cell (Uma et al., 2011). Moreover, plants can mount appropriate defensive responses triggered by the perception of physical pathogen contact mediated *via* plasma membrane-bound and intracellular immune receptors that recognize pathogen-derived elicitors or by indirect alteration of host targets (Zhang and Coaker, 2017; Kourelis and Van Der Hoorn, 2018).

Furthermore, plant-derived antimicrobial peptides and other compounds can inhibit pathogens by directly detoxifying or inhibiting virulence factor activity (Ahuja et al., 2012). Plants also initiate RNA silencing or RNA interference (RNAi) processes that detect invasive viral pathogens and cut targeted viral RNA (Rosa et al., 2018). However, pathogens have in turn evolved effective counter-strategies that enable them to circumvent host plant defensive responses. For example, numerous fungal and bacterial pathogens have been found to release cell wall-degrading enzymes (Kubicek et al., 2014), whereas when within the host cytoplasm, certain pathogen-derived effectors (Franceschetti et al., 2017) can inhibit host defenses or promote susceptibility. Moreover, it has been established that almost all plant viruses have developed RNAi inhibitors to counter RNAi-based host defense responses, as in the case of viruses that can also hijack the host RNAi system to silence host genes, thereby enhancing viral pathogenicity (Wang et al., 2012).

These host–microbe interactions, thus, provide important clues for disease resistance-targeted genetic engineering (Dangl et al., 2013). For instance, genes encoding proteins that break down mycotoxins (Karlovsky, 2011), inhibit enzymic cell wall degradation, or species of nucleic acid that can isolate inhibitors of the RNA virus (Wang et al., 2012) can be inserted into plants to reduce microbial virulence. Furthermore, plants can be engineered to synthesize and secrete antimicrobial compounds that specifically inhibit pathogen colonization (Dong and Ronald, 2019), whereas by targeting viral RNA for degradation, plant RNAi mechanisms can be manipulated to confer high viral immunity (Rosa et al., 2018). In addition, to enhance the robustness and widen the spectrum of disease tolerance, natural or edited immune receptors that recognize different pathogen strains can be inserted individually or in combination (Fuchs, 2017), and basic defense hub regulatory genes can be reprogrammed for the fine-tuning of defense responses (Pieterse and Van Loon, 2004). Similarly, genetic engineering can be used to generate host bait proteins that trap pathogens, thereby altering pathogen identification specificity (Malnoy et al., 2016).

For an additional detailed summary of the aspects of gene-edited disease-resistant plants, please refer to the review article previously published by Cook et al. (2015). In this review, we cover recent developments in the engineering of plant resistance to microbial pathogens based on the molecular mechanisms underlying plant–pathogen interactions and describe recent biotechnological advances. In the following sections, we also provide an overview of the breakthroughs in plant genetic engineering aimed at enhancing disease resistance and highlight some of the techniques that have proved promising in field trials.

## ZFN, TALEN, and CRISPR-Cas Genome-Editing Techniques

**Figure 1:**
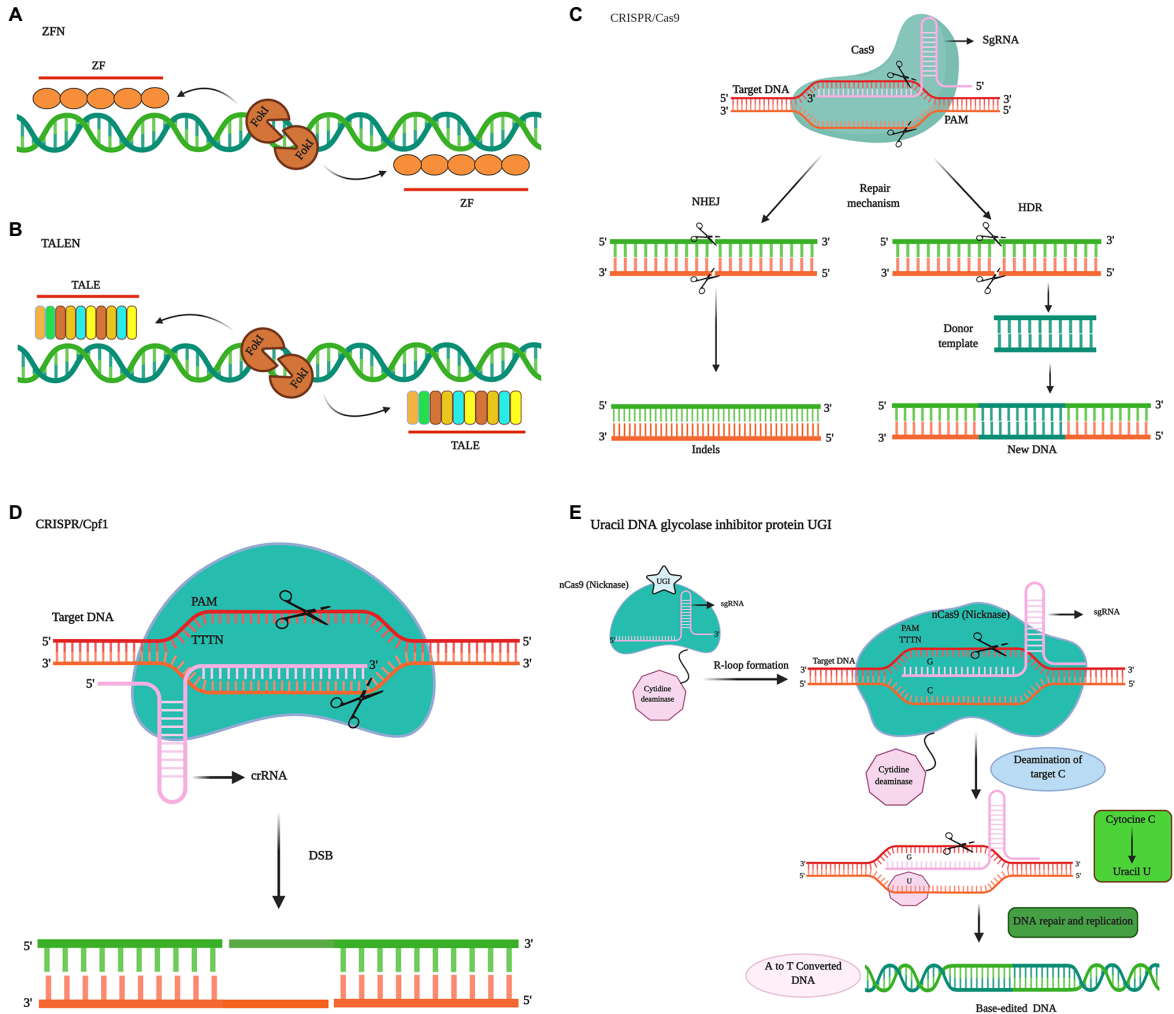
Comparison of different widely used genome editing systems in plants. Site-specific techniques, such as those based on zinc finger nucleases (ZFNs; **A**) and protein-dependent DNA cleavage systems, such as those based on transcription activator-like effector nucleases (TALENs; **B**). The two sequence-specific zinc finger proteins flank the target sequence, and *FokI* nuclease follows the C terminus of each protein. Zinc finger proteins are used to direct *FokI* dimers to target the specific DNA sites to be cleaved. TALENs consist of two sequence-specific TALEN proteins arranged on the target sequence, with *FokI* nuclease followed by the C terminus of each protein. The TALEN protein directs *FokI* dimers to the target specific DNA sites to be cleaved. **(C)** Illustration of a cluster of the regularly spaced short palindrome repeats (CRISPR)/CRISPR-associated nuclease 9 (Cas9) system. CRISPR-Cas induces double-strand breaks (DSB) in the DNA strand. CRISPR RNA (crRNA) directs the Cas9 protein. Trans-activating CRISPR RNA (tracrRNA) is used to stabilize the structure and activate Cas9 to cut the target DNA. Single guide RNA [sgRNA (Gulabi)] recognizes the target gene, and then the Cas9 protein (green) cuts the two strands of the target DNA at the RuvC and His Asn-His (HNH) domains. The cutting of DNA by Cas9 is dependent on the presence of spacer adjacent motif (PAM) sequences. A 20-base sgRNA defines the binding specificity. In DNA, there are two mechanism of double-strand break (DSB) repair, namely, homology-directed repair (HDR), which is activated in the presence of a template and leads to knock-in or gene replacement, and non-homologous end joining (NHEJ), which is not sufficiently precise and leads to permanent gene knockout. **(D)** The Cpf1 system. Cpf1 is a two-component RNA programmable DNA nuclease related to CRISPR. The PAM of Cpf1 is TTTN. Cpf1 cuts the target DNA and introduces a DSB, a 5-nt staggered cut at the 5′ end of the T-rich PAM. CRISPR-Cas9 nickase introduces breaks only in the strand complementary to sgRNA. The double nickase with two sgRNAs introduces a staggered DSB into the DNA, after which HDR repairs the DSB. Uracil DNA glycosylase inhibitor (UGI) protein prevents removal of uracil from the DNA and subsequent repair pathways and contributes to increasing the frequency of mutations.

Genome editing entails site-specific genome targeting, which is used to modify the genomic DNA of plant or animal cells with high precision and efficiency. Here, we compare three of the most widely used genome editing technologies. Among these, zinc finger nuclease (ZFN)-based modification, which is based on the use of programmable nucleases, is considered the first breakthrough in the field of genome engineering (Chandrasegaran and Carroll, 2016; Figure 1). The transcription activator-like effector nuclease (TALEN) editing using the bacterial transcription activator-like effector (TALE) is known to expand the potential utility of genome editing (Figure 1). However, the most recently developed clustered regularly spaced short palindrome repeats (CRISPR)-Cas system has attracted the most remarkable attention from researchers worldwide, on the account of its simplicity, ease of use, high efficiency, and ability to allow transgene production (Mali et al., 2013). In different organisms, including plants, application of the CRISPR-Cas9 system has rapidly surpassed the ZFN and TALEN systems (Mali et al., 2013). Unlike ZFN and TALEN systems, CRISPR-Cas9, a system that uses protein motifs for target recognition, relies on DNA/RNA recognition to generate double-strand breaks (DSBs). CRISPR-Cas9 can be considered superior to ZFNs and TALENs in the following respects: (i) simplicity of target design, (ii) efficiency of Cas9 protein and gRNA, (iii) the ease with which simultaneous targeted mutations can be generated in multiple genes (Ma et al., 2015; Malzahn et al., 2017), and (iv) vector design, given difficulties in the usability and access to developed bioinformatics tools (Ahmad et al., 2020). However, despite the multiple advantages of the CRISPR-Cas technology and significant developments to date, the technology still warrants further improvements.

Since its introduction, in recent years, the CRISPR system has been undergoing continual modifications, such as CRISPR-Cas12a (Shimatani et al., 2017) and base editing tools (Bharat et al., 2019; Butt et al., 2020), for easier use and adaptability in response to different constraints. The developed SpCas9 variant can target the expanded NGN protospacer adjacent motif (PAM), and the enzyme has been optimized to produce a near-PAMless SpCas9 variant, referred to as SpRY (NRN > NYN PAM), with SpRY nuclease and base editing variants being able to target almost all PAMs (Walton et al., 2020). Currently, the low efficiency of the homologous recombination pathway (knock-in/gene replacement) and the similar low efficiency of the transformation of homologous donor sequences in plant cells have contributed to the complexity and poor efficiency of knock-in mutations (Lu et al., 2020). Consequently, an efficient gene knock-in procedure in plants based on CRISPR-Cas-mediated homologous recombination is still required. Furthermore, the current CRISPR-Cas9 system has minimal effects with respect to the control of both RNA and DNA viruses, thereby highlighting the need to develop a useful and acceptable CRISPR system to overcome these current limitations. In this regard, recent findings have indicated that Cas13 proteins (Cas13a, Cas13b, and Cas13c) have considerable potential applicability as robust RNA regulators of RNA viruses (Abudayyeh et al., 2017; Zhang et al., 2018a). For example, CRISPR-Cas13a has been shown to confer RNA virus resistance in both monocot and dicot plants (Zhang et al., 2019a). Targeted-site gene editing has also been performed to design eIF4E resistance alleles that play important roles in virus resistance (Bastet et al., 2018, 2019).

Recently, a new genome editing technology, “prime editing,” has been developed, which can be employed to perform different types of editing, such as particular base-to-base transfers, including all transformation (C → T, G → A), (A → G and T → C), and transverse (C → A, C → G, G → C, G → T, A → C, A → T, T → A, and T → G) mutations, along with small-scale insertion/deletions, without causing DNA double-strand breakage. Given that the prime editing system has sufficient versatility to complete specific forms of genome editing, these new developments have considerable potential. Moreover, it can be modified for different purposes (including crop production, resistance to abiotic and biotic stresses, and crop plant quality improvement; Yasmin et al., 2020; Ayaz et al., 2021a). Moreover, the Cas12b (C2c1) system has recently been successfully applied for editing genomes in cotton plants to enhance the resistance to high temperatures, thereby paving the way for the development of varieties that can be cultivated under heat stress conditions (Wang et al., 2020). Consequently, by facilitating sustainable agricultural crop production, application of the CRISPR system is predicted to make a significant contribution to overcome food scarcity and ensure global food security.

## Resistance Genes Deployed for Broad-Spectrum Durable Resistance

During the early 1940s, innovative genetic studies examining the plant–pathogen interaction between flax and the flax rust fungus *Melampsora lini* were conducted (Flor, 1956), which contributed to the development of the “gene-for-gene” theory, proposing that in plant–pathogen interactions, a host plant resistance (*R*) locus matches an avirulence factor (Avr) in the pathogen (Flor, 1971). The theory maintains that as long as the *R* gene and the homologous Avr occur simultaneously, interactions between the plant and pathogen are incompatible, and the host has complete pathogen resistance (Flor, 1971). At the beginning of the 20th century, the efficacy of *R* gene-mediated resistance was first established in wheat (*Triticum* sp.) by the British scientist Rowland Biffen (Biffen, 1905). Subsequently, a number of different *R* genes have been identified, emulated, and introduced into several different varieties related to the same species, then introduced into other species of the same genera (Song et al., 1995), and ultimately across genera (Tai et al., 1999). For example, rice (*Oryza sativa*) plants expressing an *R* gene (*Rxo1*) derived from maize (*Zea mays*) were found to show resistance to bacterial streak in a laboratory environment (Hussain et al., 2019).

Similarly, multi-year trials have revealed that tomatoes (*Solanum lycopersicum*) expressing the pepper *Bs2 R* gene maintained durable field resistance to bacterial spot (*Xanthomonas* sp.) disease (Horvath et al., 2015; Kunwar et al., 2018), and under field conditions, transgenic wheat expressing different alleles of the wheat resistance locus *Pm3* showed race-specific resistance against stem rust (*Puccinia graminis* f. sp. *tritici*; Brunner et al., 2012). Furthermore, the overexpression of *R* genes, such as *Rpi-vnt1.1* or *RB* from wild potato, has been shown to confer durable resistance to potato late blight (*Phytophthora infestans*) in commercial potatoes (Jones et al., 2014; Ayaz et al., 2021b). Notably, to date, genetically modified transgenic potato overexpressing the *Rpi-vnt1.1* gene, which has enhanced resistance to potato late blight, is the only crop that has been approved for commercial use (Dong and Ronald, 2019).

Given that pathogens have the capacity to evade detection based on host *R* genes (Jones and Dangl, 2006), the disease resistance conferred by a single *R* gene typically lacks durability under field conditions, as pathogens can evolve alternative virulent forms *via* Avr gene mutation. Consequently, to obtain broad-spectrum disease resistance and thereby ensure long-lasting field resistance, multiple *R* genes are generally introduced simultaneously, a procedure referred to as gene stacking (Fuchs, 2017; Mundt, 2018). Resistance based on the stacking of *R* genes is anticipated to be both broad-spectrum and durable, given that pathogen strains are unlikely to overwhelm the resistance conferred by multiple *R* genes.

**Figure 2:**
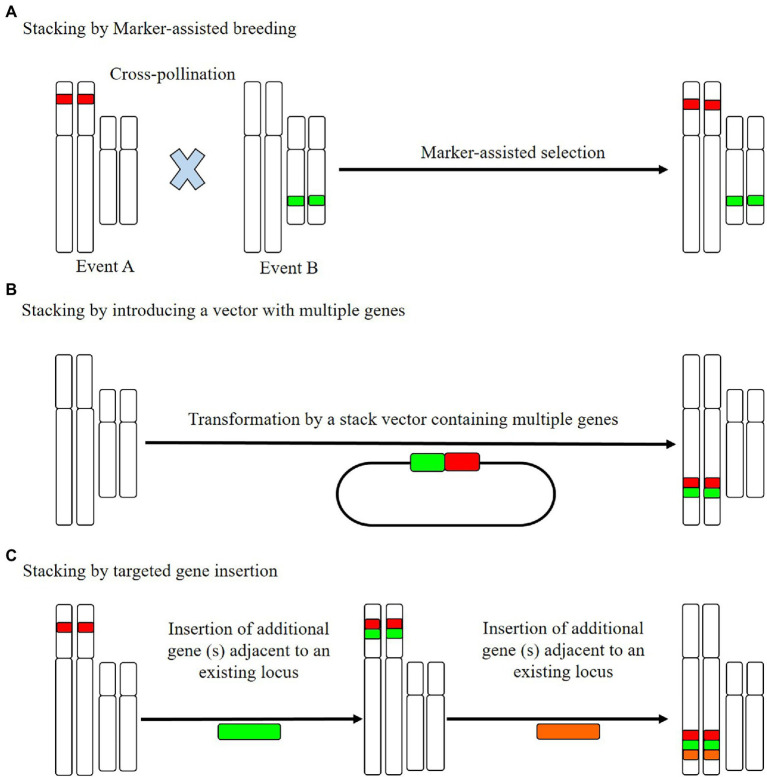
The resistance (*R*) gene stacking method. **(A)** Cross-pollinated individuals, based on established trait loci, stack marker-assisted breeding and combine the combined trait loci for marker-assisted selection of the offspring. **(B)** A single transgene stacking event can be completed by integrating multiple genes into a single stack vector and introducing these simultaneously. **(C)** Targeted insertion stacking aims to insert new genes near established loci. To stack a large number of genes, this process can be repeated. The stacked genes in B and C are genetically related, such that they can be readily inserted at a single site.

A well-established methodology for stacking *R* genes at pre-existing *R* loci is cross-breeding (Figure 2), using which, breeders can identify and select the desired progeny with requisite *R* genes, based on marker-assisted selection (Das and Rao, 2015). An example is the bacterial blight pathogen of rice (*X. oryzae* pv. *oryzae*), a lethal and devastating disease in some areas of Africa and Asia (Niño-Liu et al., 2006), for which cross-breeding has been performed to introduce three stacked *R* genes (*Xa21*, *Xa5*, and *Xa13*) and was subsequently established to contribute to resistance against this disease. These three stacked genes have also been cloned and introduced into Jalmagna (a deep-water rice cultivar; Pradhan et al., 2015), which was found to show significant durable resistance against eight *X. oryzae* isolates under field conditions (Pradhan et al., 2015).

However, despite the impressive results obtained using stacked resistance genes, the selection process can be excessively time-consuming and laborious when selection is based on the identification of a large number of loci. As a more viable alternative to gene stacking, researchers can assemble several *R* gene cassettes into a plasmid and then introduce the entire *R* gene cluster into specific genetic site *via* plant transformation (Que et al., 2010; Solangi et al., 2021). In this way, all *R* genes are inherited as a single genetic locus, thereby minimizing the time needed for selection, as illustrated by the molecular stacking of three broad-spectrum potato late blight *R* genes (*Rpi-blb3*, *Rpi-sto1*, and *Rpi-vnt1.1*) *via Agrobacterium*-mediated transformation of a susceptible potato cultivar (Zhu et al., 2012). Under controlled greenhouse conditions, this tri-transgenic potato cultivar simultaneously expressing the aforementioned three resistance genes was found to be characterized by wide-spectrum resistance, which is equivalent to the development of strain-specific resistance conferred by each of the three *Rpi* genes (Zhu et al., 2012). Similarly, *Agrobacterium*-mediated transformation has been used to simultaneously introduce *Rpi-vnt1.1* and *Rpi-sto1* into three distinct varieties of potato *via* a single DNA fragment insertion (Jo et al., 2014), which was found to confer broad-spectrum resistance to late blight. Notably, apart from the *R* genes, no external DNA, for example, in the inserted DNA fragment or a selectable marker gene, were inserted, which is considered to be advantageous with respect to transgene regulation (Jo et al., 2014). In the case of both the double- and triple-gene-stacked potatoes described here, resistance was verified under field conditions. Moreover, it has been established that the appropriate spatio-temporal deployment of potato cultivars containing *R* genes against late blight can minimize fungicide usage by more than 80% (Haverkort et al., 2016).

Similar to the aforementioned potato cultivars, a recent study conducted on African highland potato varieties in Uganda showed that the molecular stacking of three *R* genes (*Rpi-vnt1.1*, *RB*, and *Rpi-blb2*) was observed to confer durable field resistance to the potato late blight pathogen (Ghislain et al., 2019). In addition, the yield of these modified potato cultivars was established to be three times higher than the national average. These findings accordingly serve to emphasize that the gene stacking approach not only confers durable field resistance but has no detrimental impact with respect to crop yields (Ghislain et al., 2019). Moreover, these studies highlight the simplicity and efficacy of the molecular stacking used to confer broad-spectrum disease resistance in important vegetatively propagated crop organisms, for which more conventional breeding-based stacking is not practical (Figure 2). However, despite the notable benefits of molecular stacking, the performance of the stacked genes is highly dependent on the respective vectors (Que et al., 2010). Selection of appropriate vectors will facilitate the insertion of exogenous DNA sequences into plant genomes at specified targets and also enable the introduction of multiple *R* gene cassettes in the vicinity of an established *R* gene cluster (Ainley et al., 2013). In this regard, the latest innovations in genome engineering have contributed to the development of the targeted insertion of DNA segments with desired features, which can be used to incorporate diverse traits in complex crop species (Ramu et al., 2016; Voytas, 2017).

The field of genome engineering is undergoing continuous evolution and is currently in a phase of heightened activity and frequent groundbreaking developments. We can accordingly anticipate a continuous stream of new innovations that will contribute to enhance the efficiency of targeted insertion and reduce the size of inserted DNA fragments, with applications in numerous plant species. Moreover, further developments and breakthroughs in specific gene insertions will provide new opportunities to stack larger numbers of *R* genes and engineer broad-spectrum viral resistance by altering a single locus, which not only offers convenience with respect to breeding but also confers durable disease resistance.

## CRISPR-Cas-Induced Mutations and Enhanced Biotic Resistance Based on Transgene-Free Methods

Although sequence-specific nucleases, such as CRISPR-Cas, play an important role in transgene manipulations (for example, when the T-DNA in *Agrobacterium tumefaciens* provides CRISPR-Cas9 and sgRNA), the induced mutations may be genetically independent of the site of construct integration. This means that even if the CRISPR process involves an intermediate transgenic state, it is possible to produce genetically modified-free crops by merely isolating the mutation site from the insertion site using CRISPR-Cas techniques. In this regard, PCR can be used to characterize individual plants and determine the sequences of entire genomes, which is beneficial with respect to assessing mutations at the target site and also in identifying potential off-target mutations (Kim and Kim, 2016). Although in the United States, CRISPR-modified crops have been evaluated *via* product-based legislation and are not covered by genetically modified organism principles (Waltz, 2018), the current situation in Europe tends be more complex, in that regulation in European countries is dependent on process-based legislation (Sprink et al., 2016). However, given that it is practically impossible to trace back to the initial induction of a small mutation (whether it be introduced naturally; chemically by ethyl methanesulfonate; *via* X-ray radiation, oligonucleotide-directed mutagenesis, or TALENs, or physically by CRISPR-Cas), the attempts to regulate CRISPR-mediated mutations in the European Union would appear to be unrealistic (Urnov et al., 2018). In this context, transfection of plant protoplasts with the ribonucleoprotein complex of any genetically modified intermediate consisting of Cas9 protein and sgRNA is considered a theoretical solution (Metje-Sprink et al., 2019). Successful attempts to circumvent problems associated with the genetically modified status of edited plant using “DNA-free” systems have previously been reported for a number of species, including *Chlamydomonas reinhardtii* (Baek et al., 2016), *Zea mays* (Svitashev et al., 2016), petunia (Subburaj et al., 2016), wheat (Liang et al., 2017), apple (*Malus domestica*) and grapevine (*Vitis vinifera*; Malnoy et al., 2016), lettuce (*Lactuca sativa*), rice, tobacco (*Nicotiana tabacum*), and *Arabidopsis thaliana*, and can contribute to further reductions in possible off-target mutations (Table 1).

**Table 1:** The application of diverse techniques for the improvement of different agronomic and disease resistance traits in crops species.

Crop species	Technology	Target genes	Result/Trait improvement	Reference
*Triticum aestivum*	TALEN	*TaMLO*	Powdery mildew resistance	Wang et al., 2014
*Manihot esculenta*	CRISPR-Cas9	*MenCBP-1/2*	Cassava brown streak virus resistance	Gomez et al., 2019
*Hordeum vulgare*	CRISPR-Cas9	*HvMorc1*	*Blumeria graminis*/*Fusarium graminearum* resistance	Kumar et al., 2018
*Gossypium hirsutum*	CRISPR-Cas9	*Gh14-3-3d*	*Verticillium dahlia* resistance	Zhang et al., 2018b
*Nicotiana benthamiana*	CRISPR-Cas9	*IR*, *C1*	Cotton leaf curl multan virus resistance	Yin et al., 2019a
*Brassica napus*	CRISPR-Cas9	*BnCRT1a*	*Verticillium longisporum* resistance	Pröbsting et al., 2020
*Triticum aestivum*	CRISPR-Cas9	*TaNFXL1*	*Fusarium graminearum* resistance	Brauer et al., 2020
*Citrullus lanatus*	CRISPR-Cas9	*Clpsk1*	*Fusarium oxysporum* resistance	Zhang et al., 2020a
*Oryza sativa*	CRISPR-Cas9	*OsCul3a*	*Xanthomonas oryzae/Magnaporthe oryzae* resistance	Gao et al., 2020
*Oryza sativa*	CRISPR-Cas9	*OsPi21*, *OsXa13*	*Magnaporthe oryzae*/*Xanthomonas oryzae* resistance	Li et al., 2019a
*Solanum tuberosum*	TALEN	*ALS*	Herbicide resistance	Butler et al., 2016
*Zea mays*	ZFN	*PAT*	Herbicide resistance	Schornack et al., 2006
*Gossypium hirsutum*	CRISPR-Cas9	*ALARP*	Cotton fiber development	Sander and Joung, 2014
*Manihot esculenta*	CRISPR-Cas9	*elF4E isoforms nCBP-1 & nCBP-2*	*Cassava brown streak virus*	Gomez et al., 2019
*Oryza sativa*	CRISPR-Cas9	*SWEET11*, *SWEET13* and *SWEET14/promoter*	*Xanthomonas oryzae* pv. *Oryzae* resistance	Oliva et al., 2019
*Oryza sativa*	CRISPR-Cpf1	*OsEPFL9*	Regulation of stomatal density	Yin et al., 2019b
*Citrus*	CRISPR-Cas9	*Citrus CsLOB1*	*Xanthomonas citri* subsp. *Citri* resistance	Jia et al., 2017
*Solanum lycopersicum*	CRISPR-Cas9	*Solyc08g075770*	*Fusarium oxysporum* f. sp. *lycopersici*,	Prihatna et al., 2018
*Cucumis sativus*	CRISPR-Cas9	*elf4E/cds*	Resistance to CVYV, ZYMV, and PRSMV	Chandrasekaran et al., 2016
*Glycine max*	CRISPR-Cas9	*GmF3H1/2*, *FNSII-1*	Soybean mosaic virus	Zhang et al., 2020b
*Malus domestica*	CRISPR-Cas9	*PDS*, *TFL1*	Albino phenotype, early flowering	Charrier et al., 2019
*Vitis vinifera*	CRISPR-Cas9	*VvPDS, MLO-7*	Albino phenotype	Nakajima et al., 2017
*Musa* spp.	CRISPR-Cas9	*ORF1, 2, 3* and *IR of BSV*	Resistance against Banana streak virus	Tripathi et al., 2019
*Arabidopsis thaliana*	CRISPR-Cas9	*eIF4E*	Transgene free resistant against Clover yellow vein virus	Bastet et al., 2019
*Citrullus lanatus*	CRISPR-Cas9	*Clpsk1*	Resistance to *Fusarium oxysporum f*. sp. *niveum*	Zhang et al., 2020a
*Oryza sativa*	CRISPR-Cas9	*EBEs of OsSWEET14*	Resistance to *Xanthomonas oryzae* pv. *oryzae*	Zafar et al., 2020
*Capsicum annuum*	CRISPR-Cas9	*CaERF28*	Anthracnose disease resistance	Mishra et al., 2021

Genes encoding certain susceptibility factors are also considered potentially useful targets for manipulation, as resistance can already be increased based on a simple knockout (Zaidi et al., 2018). For example, in some plant species, it has been established that plants harboring recessive MLO alleles are characterized by resistance to powdery mildew, as demonstrated by the modification of MLO in tomatoes (Nekrasov et al., 2017). TALENs and genome editing provide a basis for the so-called new breeding technology involving CRISPR-Cas editing, which has been used to mutate all six wheat MLO alleles, an in the targeted inactivation of existing alleles and genomes in a number of polyploidy crops, resulting in enhanced resistance to the *Blumeria graminis* f. sp. *tritici* fungal pathogen (Wang et al., 2014; Table 1). In rice, resistance to *X. oryzae* pv. *oryzae* has been obtained by mutating *SWEET* resistance genes or by engineering genes their promoters with a bacterial pathogen (Oliva et al., 2019; Xu et al., 2019).

Enhanced tolerance can also be achieved by deleting, rewriting, or inserting *cis* elements in the promotors of susceptibility or resistance genes. The *cis* elements targeted by effectors in susceptibility genes can be eliminated by taking advantage of the fact that the gene remains intact and can still perform its normal plant growth functions. Furthermore, TALENs have been used to modify *cis* elements in the promoter of the *OsSWEET14* gene in rice, which targets the rice blast *AvrXa7* gene. Although this reduction in *cis*-factor activity leads to fewer extreme disease symptoms, it is achieved *via* a TALEN-based method, which promote bacteria (Li et al., 2012). Although rewriting *cis* elements *via* homology-directed repair (HDR) using repair templates to modify the promoter is considered more difficult, it does, nevertheless, ensure that indels do not interfere with the spatial distribution of *cis* elements in promoters, which is necessary for the maintenance of appropriate host plant gene regulation. Moreover, the insertion of new *cis* elements into the promoters of defense-related genes can contribute to enhance the expression in response to new stress signals/pathogens.

**Figure 3:**
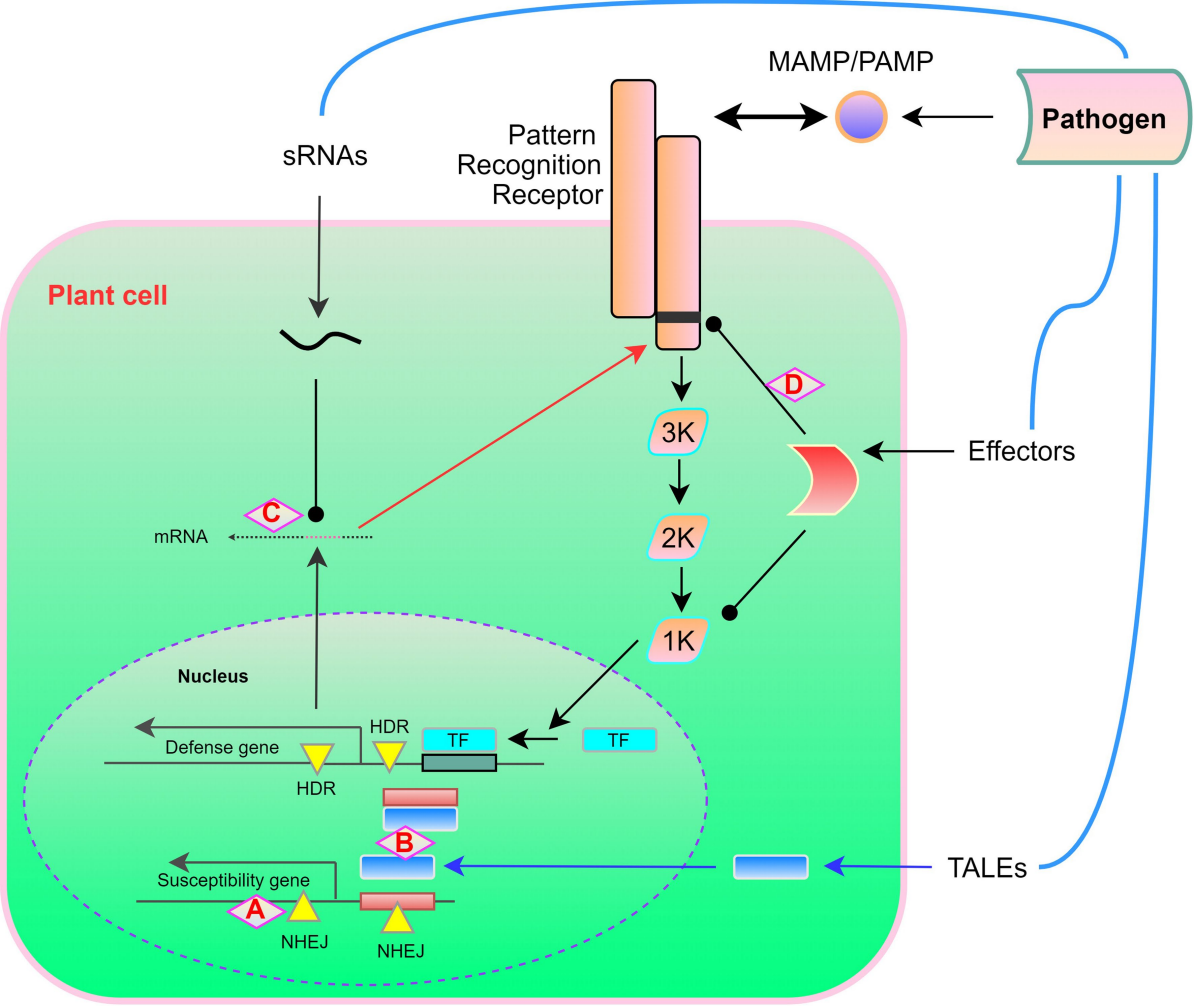
A potential target for applying CRISPR-Cas for engineering of “non-transgenic” disease resistance in plants. In response to pathogen recognition, the plant-induced defense response involves a MAP kinase phosphorylation cascade, which leads to the activation of transcription factors and the expression of defense-related genes. **(A)** Open reading frame disruption by non-homologous end joining (NHEJ) causes frameshift mutations in susceptibility genes. **(B)** NHEJ eliminates *cis* elements to prevent transcription activator-like effector (TALE) activation of susceptibility genes and homology-directed repair (HDR) introduces *cis* elements, which promotes the TALE-triggered activation of defense genes. **(C)** Rewriting of transboundary RNAi *via* HDR-mediated pathogen siRNA targeting. **(D)** HDR-mediated coding sequence rewriting replaces the effectors produced by pathogens for amino acid residues required for protein recognition, thereby preventing, for example, cleavage or modification. The figures are adopted with modification from those presented by Schenke and Cai (2020).

However, a double mutation in the BIK1 protein (G230A/D231A) has been found to result in an flg22-induced defense signaling dominant-negative effect, thereby indicating that these two amino acid residues are functionally essential (Zhang et al., 2010). Any of the hyper-phosphorylated amino acids may be substituted to dampen effector-mediated host susceptibility. The typical role of RIN4 in suppressing the immune response involves FLS2-activated serine 141 phosphorylation, and threonine 166 phosphorylation is mediated by the AvrB effector (Chung et al., 2014). Although AvrB has been observed to reduce pathogen-induced callus deposition in wild-type plants, this inhibitory effect is eliminated in mutant plants that express non-phosphorylated RIN4 (T166A). These findings, thus, emphasize the necessity of taking multiple factors into consideration. Generally, however, to enhance plant resistance, it should be feasible to substitute effectors with amino acids. Targeted-base editing of catalytically dead Cas9, such as the cytosine deaminase domain, has the potential to become a powerful tool, enabling molecular biologists to share unique amino acids that disable effectors and target interactions. These potential developments in CRISPR-Cas-based resistance engineering are also summarized in Figure 3. In all these cases, it is worth noting that resistance is race-specific against the pathogen that has deployed the corresponding effector. Some host genome modifications (mutation stacking) can also contribute to the development of new pathogen-resistant crop varieties and more efficient forms of plant immunity.

## Transgenic Techniques That Use CRISPR-Cas Systems to Enhance Disease Resistance

Genome editing also enables the transfer of heterologous genes involved in resistance pathway mechanisms. The distinction between this approach and T-DNA transfer methods is that the space between identified highly expressed protection genes can be precisely selected using CRISPR-Cas-HDR in response to the pressure exerted by pathogen strains. This alternative approach will predictably improve regulatory accessibility and increase expression of the genes conferred by transfer resistance. By using this strategy, it will also be possible to reduce the potential negative effects on other host genes in the vicinity of the site of integration (Schenke and Cai, 2020). Given that the flanking regions are known, string integration can typically detect positive events in such genomic regions without the necessity of using selectable markers, and thus, it is possible to perform PCR-based locus amplification and sequencing. The first generation of genetically modified organisms has often been criticized, notably on account of certain possible side effects attributable to the random selection of genetically modified genes and because antibiotic resistance genes were necessary for the detection of positive integration events (Gepts, 2002). These drawbacks can now be overcome, although it remains important to control the introduction of foreign DNA, a time-consuming and cost-intensive procedure, which normally has low consumer acceptance (Waltz, 2018).

CRISPR-Cas that can be used to transfer HDR-mediated resistance genes into the actively expressed chromatin regions of susceptible hosts to confer stress tolerance can contribute to the breeding of resistant varieties. This feature acquisition technique can be simplified using CRISPR-Cas genome editing. For example, it is possible to jointly regulate *R* genes closely linked in a head-to-head configuration to ensure that they can function in tandem to impart resistance. The expression of *R* genes in such regulatory modules will reduce the probability of imbalance and further minimize fitness costs (Karasov et al., 2017). In this regard, *R* genes, such as nucleotide-binding site leucine-rich repeat sequence (NLR) genes, typically function in pairs, including NLR accessory molecules and sensors that can recognize pathogenic effectors, although they do not control autoimmunity prevention mechanisms in the case of pathogens (Wu et al., 2017). By transferring *R* genes to crops, in which they can be expressed in response to biotic stress, pathogen resistance can be enhanced, although the number of appropriate *R* genes is limited and the identity of the interacting auxiliary NLR will need to be verified (Wang et al., 2019).

Furthermore, it has been established that dominant *R* gene-mediated drug resistance is not as stable as is recessive drug resistance and is typically confined to a minority of race-specific isolates that can readily be resolved by higher mutation rates (Kourelis and Van Der Hoorn, 2018). Therefore, achieving stronger resistance can be achieved by stacking/pyramiding different traits, and in this regard, CRISPR-Cas-induced HDR should be further encouraged (Pandolfi et al., 2017). Furthermore, pattern recognition receptor (PRR) transfer has been demonstrated to confer broader and more robust resistance in *Arabidopsis* and tomatoes (Lacombe et al., 2010; Saqib et al., 2020), banana and rice (Tripathi et al., 2014), and wheat (Manghwar et al., 2021). This is feasible owing to the maintenance of comparable immune signaling systems in monocots and dicots (Holton et al., 2015). The benefit of these approaches is that the modifications are tailored to specific pathogens, with defense responses being initiated when plants are threatened by the corresponding pathogens, thereby reducing potential losses of yield (Karasov et al., 2017). It is well established that by generating receptive chromatin regions, stress can induce a priming response, thereby promoting the more rapid expression of protective genes during secondary challenges by same stress source (Manghwar et al., 2022; Manghwar and Hussain, 2022). These genomic regions can accordingly have beneficial effects with respect to the expression of heterologous *R* genes or PRRs.

## Base Editing for Conferring Disease Resistance in Crops

Base editing technology has evolved as a revolutionary technique for genome editing in plants that is both efficient and effective. Despite being developed only recently, several papers have already been published describing the use of these techniques to enhance agronomic features and disease resistance in a range of agriculturally important crops. Base editing has shown considerable potential for trait development in rice, and this technique might also be applied in other monocot crops, such as maize, sugarcane, barley, wheat, and sorghum (Yarra and Sahoo, 2021). Base editing involves the use of a catalytically deficient CRISPR-Cas nuclease coupled to a nucleotide deaminase and, in some cases, DNA repair proteins. Using this approach, single-nucleotide variations can be engineered at specified loci in the DNA (nuclear or organellar) or RNA of both dividing and non-dividing cells. Base editors (BEs) can be divided into two categories, namely, DNA BEs, which can be used to introduce specific point mutations in DNA, and RNA Bes, which can alter single ribonucleotides. The different types of DNA BEs that are currently accessible include cytosine BEs (CBEs), adenine BEs (ABEs), C-to-G BEs (CGBEs), dual-base editing, and organellar BEs, among which, CBE and ABE, given their simplicity and efficacy in precise base editing, have been widely employed in numerous organisms, including plants, for both gene functional annotation and gene correction (Veillet et al., 2019b; Yarra and Sahoo, 2021).

### Cytidine Base Editors

**Figure 4:**
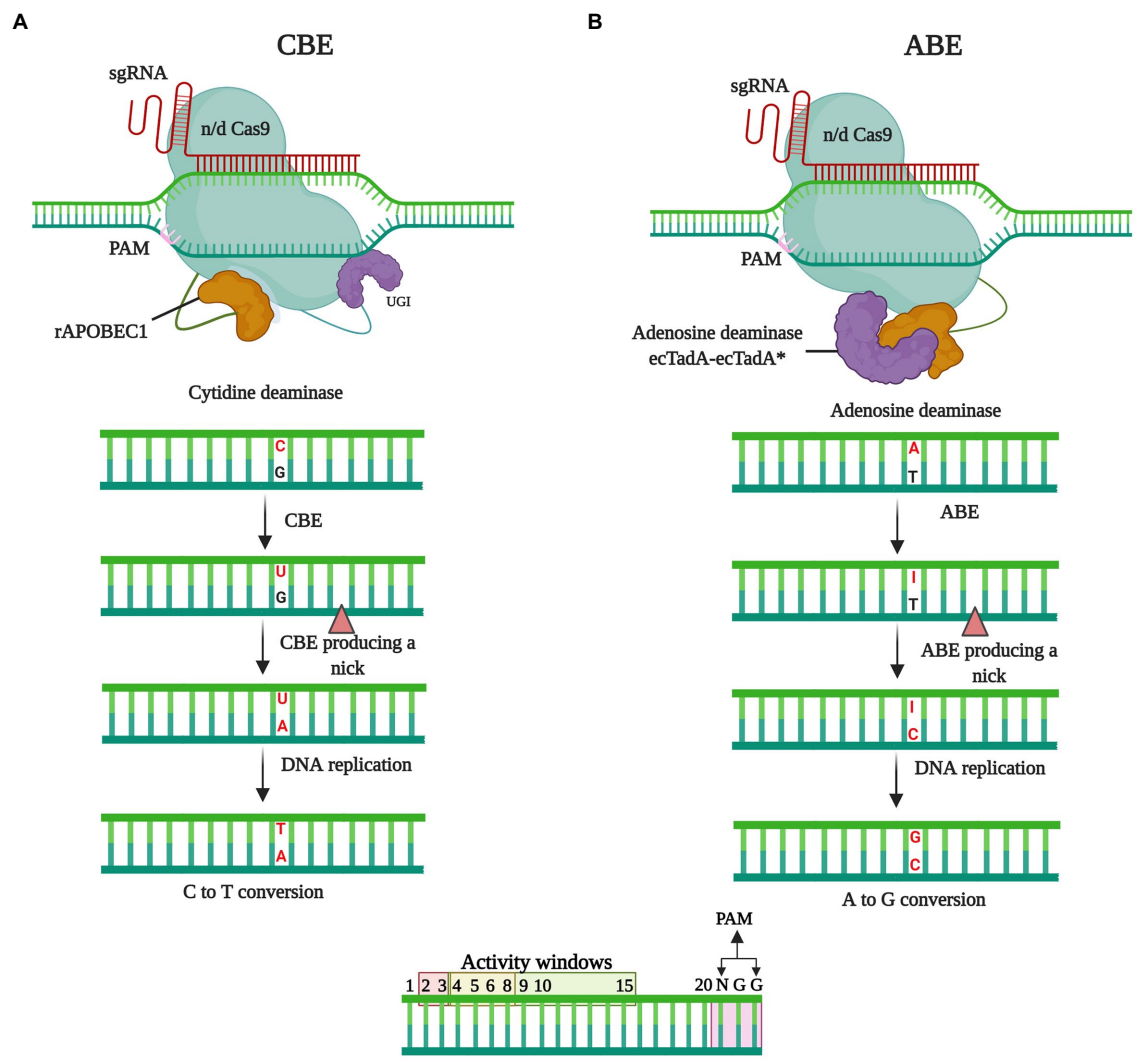
The mechanisms of base editing. In the absence of double-strand breaks (DSBs), base editing facilitates the introduction of precise point mutations at specified target sites in the genome *via* nucleotide substitution. **(A)** Cytidine deaminase base editing (CBE) is performed in conjunction with the use of an APOBEC1 cytidine deaminase, which converts C to U. Subsequently the U-G mismatch is resolved *via* cellular mismatch repair or base editing mismatch repair machinery that leads to the formation of T-A at the target locus. **(B)** The adenine base editing (ABE) leads A-T to G-C substitution. After recruiting to the targeted genomic locus, the ABE delaminates targeted A base to I (inosine) leading to I-T base pairing. The cellular mismatch repair mechanism or DNA replication resolves the I-T, forming G-C base pairing.

Cytidine base editors, the first-developed type of DNA base editor, are used to facilitate the conversion from C-G to T-A (Komor et al., 2016), and in two seminal investigations, CBEs with diverse topologies incorporating a Cas9 nickase (nCas9, for example, with a D10A mutation) coupled to cytidine deaminase and uracil glycosylase inhibitor (UGI) were described (Nishida et al., 2016; Figure 4A). Similar to the canonical CRISPR–Cas systems, CBEs are guided to target genomic region by a sgRNA. After binding to the target DNA, an sgRNA–CBE complex forms a single-stranded DNA R-loop (Jiang et al., 2016), and CBE cytidine deaminase, which catalyzes the hydrolytic deamination of an exposed cytosine, gains access to this non-target single-stranded DNA. C-to-T base editing is mediated *via* deamination and subsequent cellular mismatch repair. This process tends to be hampered by uracil (U) base excision repair (BER), which either regenerates the original base pair or results in indels (Porto et al., 2020). However, the action of UGI subverts BER and increases the likelihood of C-to-T editing. Although antibiotics are commonly used to select transformants, it may be difficult to detect base-edited cells in a population. To circumvent this constraint, a surrogate reporter system based on the repair of a faulty hygromycin-resistance gene has been constructed in plants (Xu et al., 2020b). The editing of cytidine bases for enhancing crop agronomic traits and disease resistance has already found application in a range of plant species, including rice (Hua et al., 2019; Ren et al., 2021; Yarra and Sahoo, 2021); *Arabidopsis* (Chen et al., 2017; Li et al., 2019b); wheat (Zong et al., 2017; Zhang et al., 2019b); maize (Zong et al., 2017); potato and tomato (Veillet et al., 2019a; Hunziker et al., 2020); watermelon, cotton, soya bean, apple, and pear (Zhao et al., 2018; Cai et al., 2020; Qin et al., 2020; Malabarba et al., 2021); and strawberry and rapeseed (Xing et al., 2020; Cheng et al., 2021).

### Adenine Base Editors

On the basis of the characterization of CBEs, it was assumed that a combination of adenine deaminase and nCas9 would give rise to ABEs, which could be used to convert an A-T base pair to a G-C base pair. However, none of the naturally occurring adenine deaminases have been found to work with DNA (Gaudelli et al., 2017; Molla et al., 2021; Figure 4B). However, Gaudelli et al. developed a single-stranded DNA-specific transfer RNA (tRNA) adenosine deaminase (TadA) variant using directed evolution and protein engineering (Gaudelli et al., 2017), into which mutations were introduced to generate an engineered version (TadA*). Given that TadA catalyzes deamination as a dimer, a heterodimeric protein containing a non-catalytic wild-type TadA monomer and a designed catalytic monomer (TadA*) was developed (Gaudelli et al., 2017; Rees and Liu, 2018). The fusion of this heterodimer (TadA–TadA*) with nCas9 was accordingly found to yield ABEs that can efficiently convert A to G in high-purity mammalian cells (Molla and Yang, 2019). In contrast to uracil excision repair, cellular inosine excision repair is comparatively weak and causes little interference with A-T to G-C conversions (Yarra and Sahoo, 2021). Consequently, no other glycosylase inhibitor protein is necessary in the development of ABEs (Molla et al., 2021). To date, ABE systems have been used to modify growth traits and disease resistance in *A. thaliana* and *Brassica napus* (Kang et al., 2018), rice (Molla et al., 2020; Yarra and Sahoo, 2021), and wheat and *Nicotiana benthamiana* (Li et al., 2018; Wang et al., 2021). Collectively, the base editing tools developed thus far have shown considerable potential for enhancing the efficiency of single-base editing, with applications for the modification of a broad range of agronomic and disease resistance traits in crops.

## Prime Editing for Plant Disease Resistance

**Figure 5:**
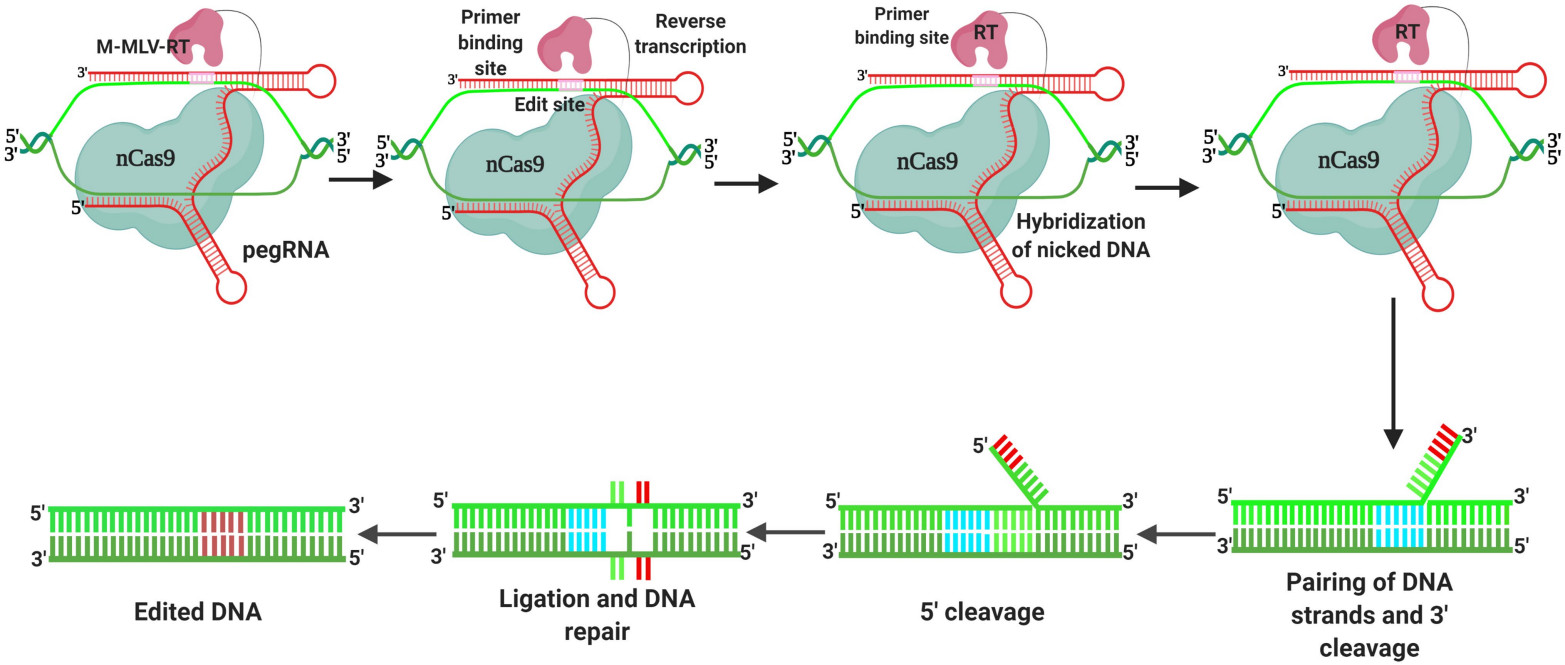
Prime editing is a recently designed base editing technique that facilitates the accurate substitution, insertion, and deletion of sequences. The fusion of Nickase Cas9 (nCas9) and reverse transcriptase (RT) is the most important element in prime editing. The intended modifications are encoded by the prime editing gRNA, which directs the nCas9-RT complex to the target gene sequence. The DNA is cut by the prime editor, which is then hybridized to the primer-binding site, resulting in reverse transcription. DNA ligation and repair are followed by base pairing of 30 or 50 flaps, resulting in DNA editing.

A pioneering genome editing approach that overcomes the issue of transversion editing was developed prior to the introduction of transversion base editors (Figure 5). The “prime editor” technique, which can be used to introduce 12 different base changes in human cells, comprises nCas9 (H840A) coupled to the reverse transcriptase of the Moloney murine leukemia virus (M-MLV RT), as well as a prime editing guide RNA (pegRNA) with a reverse transcriptase template and a primer-binding site at the 3′ end of the sgRNA. The reverse transcriptase template contains the genetic material for the desired mutation, whereas the primer-binding site joins the nCas9 (H840A)-nicked ssDNA strand (Azameti and Dauda, 2021). Following the priming of reverse transcription, the genetic information from the reverse transcriptase template is incorporated into the genome, and in response to cutting of the ssDNA approximately 3 base pairs upstream of the PAM sequence on the non-target strand, a 3-base pair extension of the pegRNA, which contains both a primer-binding site and a reverse transcription sequence (RT sequence), an appropriate polymorphism can be introduced at the target location (Anzalone et al., 2019). Although it induces base replacements and introduces a few indels at a relatively wide range of locations (+1 to +33), the primary editor is not limited by its PAM sequence (Azameti and Dauda, 2021).

To date, maize, wheat, and rice are among the crops for which prime editing systems have been developed and deployed (Hua et al., 2020; Lin et al., 2020; Xu et al., 2020a), and recently, prime editing technology has been used to generate point mutations, insertions, and deletions in the protoplasts of rice and wheat plants, achieving a regeneration of prime-edited rice plants of up to 21.8% (Lin et al., 2020). In addition, an HPT-ATG reporter has been developed in rice to generate and assess the activity of a plant prime editor 2 (pPE2) system (Xu et al., 2020a), with the authors obtaining up to 31.3% edited transgenic T_0_ plants. Using the prime editor to target *OsALS-1* and *OsALS-2*, Xu et al. (2020c) found that the predicted G-to-T and specific C-to-T substitution editing efficiencies were 1.1% (1/87) and 1.1% (1/88), respectively. In a further example of the application of this technology, Hua et al. (2020) developed the prime editor Sp-PE3 and investigated its efficacy in rice calli, achieving an editing efficiency of up to 17.1% at the targeted locations, among which, the rice endogenous acetolactate synthase (ALS) gene was edited to generate the desired G-A base transition in four of the 44 (9.1%) assessed transgenic lines, with no insertions or deletions. Interestingly, using the same editor system, no mutations were detected at the ABERRANT PANICLE ORGANIZATION 1 (APO1) locus, thereby implying that Sp-PE3 can be used to facilitate precise base substitution with varying degrees of efficacy, depending on the targeted location. Prime editors have also been used to generate mutant maize lines with double (*W542L/S621I*) mutations (Jiang et al., 2020), with 43.75% (7 of 16) of the lines transformed with *pZ1WS* also containing an edited *S621I*, and a single line found to contain homozygous mutations in both *ZmALS1* and *ZmALS2*. Attempts have also been made to engineer herbicide resistance in rice by targeting ACETOLACTATE SYNTHASE (OsALS), with a verified editing efficiency of 0.26–2% at the targeted location (Butt et al., 2020; Hussain et al., 2021), and reported development of resistant rice as a consequence of base substitutions. Furthermore, Veillet et al. (2020) used CRISPR-mediated plant prime editing to successfully target potato *StALS* genes, with an editing efficacy of 92%. Nevertheless, despite the use of orthogonal approaches, such as reverse transcriptase orthologs with differing catalytic activity, the efficacy of prime editor-based editing in plants remains limited (Lin et al., 2020; Xu et al., 2020a).

## RNAi Approaches for Conferring Disease Resistance in Plants

Plants have evolved inherent RNA interference (RNAi) mechanism, which they deploy to minimize the presence of unwanted elements and develop virus resistance (Kamthan et al., 2015; Rosa et al., 2018). RNAi functions by suppressing gene expression or neutralizing specific mRNA molecules, and the dominant nature of functional RNAi hairpins may be seen into plants. As an example of RNAi-based genome editing in plants, a gene encoding P450, an enzyme responsible for herbicide resistance, has been targeted using an RNAi expression element inserted into a CRISPR-Cas9 construct, with transgene-free genome-edited resistant plants being obtained, as determined by herbicide-based isolation phenotyping rather than PCR to identify and separate the transgene-free plants (Majumdar et al., 2017). Numerous recent studies have similarly used RNAi approaches to confer disease resistance in plants (e.g., Kuo and Falk, 2020; Liu et al., 2021).

Similar resistance may also be conferred using non-coding RNA, with miRNA expressed *via* RNA interference with polymerase II (RNAi) typically being the underlying mechanism in eukaryotic cells. Small RNAs are mobile elements that can be interchanged among plants and pathogens (Hua et al., 2018). It has been shown that pathogen-derived sRNAs target host mRNAs encoding genes associated with RNAi degradation and defense (Weiberg et al., 2013), whereas in response to pathogen attack, plants initiate the differential expression of endogenous sRNAs (Shen et al., 2014) and may even target *in vivo* factor to pathogens (Zhang et al., 2016). Thus, given that RNAi is a natural phenomenon, in order to target pathogens, it is only necessary to modify the underlying mechanisms by converting small artificial miRNAs homologous to viral genomes to small molecular miRNAs. For example, a common target is the viral coat protein or viral replication (Dong and Ronald, 2019). Moreover, stable constitutive expression of CRISPR-Cas systems can be employed to target viral genomes with adequate sgRNA, thereby generating a new immune system (Zhang et al., 2018a).

Both small sgRNA and virus-specific proteins can be modified using CRISPR-Cas technology. The mechanisms are sufficient to degrade pathogen genomes or intermediate replication in response to the viral infection of host cells. Notably, this miRNA or sgRNA should be carefully engineered such that it does not inadvertently attack the host or human nucleic acids. Given that the principal mechanisms of resistance (degradation of nucleic acids derived from important pathogens) are broadly comparable, the application of either system could be used to generate transgenic crops *via* host gene-induced silencing (HIGS) and CRISPR-Cas methods. In this regard, although counter-defensive measures (inhibitor proteins) against the host RNAi mechanisms have been identified in at least some plant viruses (Voinnet, 2005), which could nullify HIGS, these would not detrimentally influence the bacterial CRISPR-Cas system in the event of co-infection events. However, as demonstrated by the *Geminivirus* infection of cassava, it should be taken into consideration that CRISPR-Cas9 can also influence virus evolution (Mehta et al., 2019; Rybicki, 2019).

It is assumed that it would be feasible to engineer specific mutations in host defense factor coding sequences based on HDR. However, given that a sufficient number of repair templates is necessary to facilitate repair, the efficiency is naturally lower than that achieved based on non-homologous end joining (NHEJ). Given the degenerative nature of the genetic code, base triplets may be substituted without altering start sequence amino acids in the open reading frame. For example, by targeting plant defense genes, pathogen-derived siRNAs may succeed in hijacking the host RNAi machinery, thereby preventing the degradation of mRNAs encoding (trans-kingdom RNAi). The potential objectives of this approach have been established with respect to the interaction between tomato and *Botrytis cinerea*, which affects the defense signal transduction sequence MAPKKK4 (Weiberg et al., 2013), and in the interaction between *Arabidopsis* and *B. cinerea*, targeting *AtWRKY7*, *AtPMR6*, and *AtFEI2* (Wang et al., 2017). The advantage of this approach is that there is no implementation cost. Specific amino acids required for pathogen effector recognition and/or cleavage may also be altered in targeted plant proteins. For example, HopB1, which cleaves BAK1 (Li et al., 2016), can be used to cleave signaling components PBS1, PBL1, PBL2, PBL6, and BIK1 co-receptors or AvrPphB among *Pseudomonas syringae* effector proteases (Zhang et al., 2010), as even a single amino acid change in the Aβ cleavage motif prevents AvrPphB cleavage. This RNA surveillance system also functions as a powerful antiviral defense mechanism in plants.

## Potential Utility of CRISPR-Cas-Mediated Genome Editing in Sustainable Agriculture

**Figure 6:**
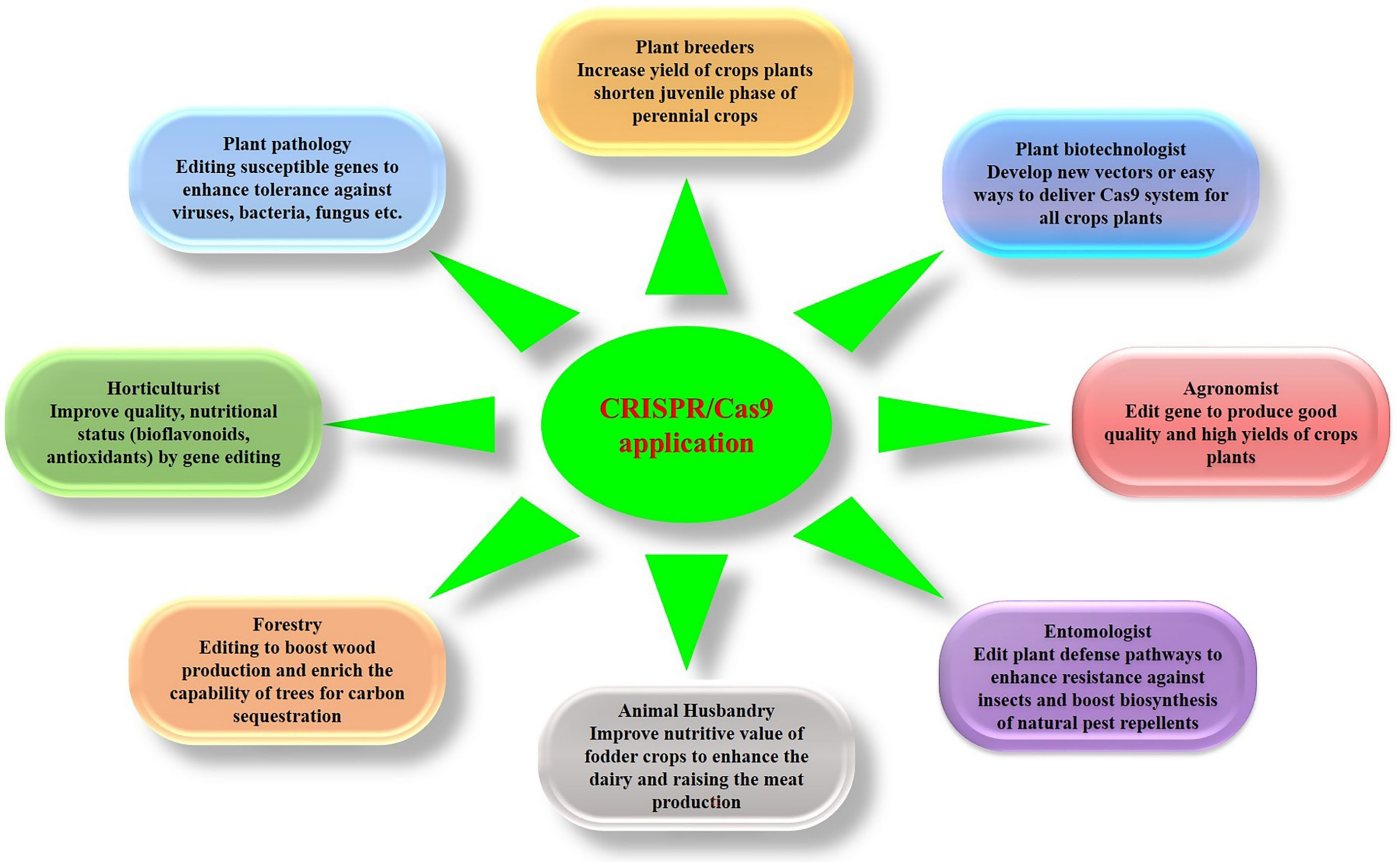
An overview of genome engineering technology and the potential use of the CRISPR-Cas system in different agriculture science disciplines.

Over the past 100 years, agricultural productivity has grown substantially as a consequence of continual technical advances (Voytas and Gao, 2014). In recent times, the advent of molecular genetic tools has ushered in a new era of genomic breeding (Figure 6), based on genetic engineering and molecular breeding (Wallace et al., 2018). Both transgenic and non-transgenic methodologies have, for a number of years, contributed to groundbreaking developments in agriculture and horticulture. However, despite the fact that transgenic crops continue to be the focus of crop improvement (Green, 2018; Nakka et al., 2019), public acceptance of these modified plants is typically limited (Voytas and Gao, 2014). To circumvent the wariness associated with the introduction of foreign genetic material, numerous genome editing approaches are currently being assessed for their utility in the development non-transgenic crops, some of which have recently been marketed (Metje-Sprink et al., 2020). In this context, the majority of the studies conducted to date have tended to focus on “proof of concept” or improving the precision and delivery of the site directed nucleases SDN (Metje-Sprink et al., 2020). Although genome editing has been used to generate a number of crops, there have yet been very few agricultural trials of these crops. Among those genome-edited crops that have reached the field trial stage are herbicide-resistant canola and flax plants. For example, in the United States, CIBUS conducted one of the first field trials for herbicide-resistant canola (Hussain et al., 2021), which, given that it is not genetically modified. Bayer Crop Science has developed a genome-edited flax with glyphosate tolerance, which, in 2019, was successfully cultivated over an extensive area of approximately 50 million acres (Hussain et al., 2021). The cultivation of such genome-edited crops, particularly those characterized by multiple resistance, can represent a key strategy for simultaneously combating numerous stresses and enhancing crop yields, while also preserving soil moisture and texture (Zhang et al., 2020c), and it is hoped that trials of a larger number of these crops are inaugurated in the coming years.

Advances in genome editing, particularly the introduction of CRISPR-Cas systems, have opened up new avenues for crop development (Han and Kim, 2019; Zhang et al., 2019c). In this regard. The editing of plant genomes has a number of notable benefits. For example, wheat plants engineered for the triple knockout of *TaMLO* not only showed resistance to powdery mildew but also showed resistance to chlorophyll (Wang et al., 2014), whereas the triple mutants obtained based on non-conservative EMS-induced *TaMLO* target mutations showed evident pleiotropic effects (Acevedo-Garcia et al., 2017). Recently, a CRISPR system has been used to alter the coding and promoter regions of the citrus canker susceptibility gene *LOB1*, thereby conferring citrus canker resistance (Jia et al., 2017; Peng et al., 2017), and the fruits of these modified plants have been marketed as a sustainably cultivated commercial product for human consumption (Waltz, 2016). Furthermore, CRISPR-Cas9 has been used to facilitate the knockout of *Ms1* in wheat lines for hybrid seed production (Okada et al., 2019). Moreover, genome editing enables the direct insertion of exogenous genes into plants to impart biotic or abiotic stress resistance (Fartyal et al., 2018). For example, phosphinothricin acetyltransferase (PAT) and bialaphos resistance (BAR) genes, initially obtained from species of *Streptomyces*, have been introduced into plants to confer glufosinate herbicide resistance (Schütte et al., 2017), whereas Han and Kim have used CRISPR-Cas9 to induce loss-of-function mutations of 5-oxoprolinase (OXP) and phosphoribosyl anthranilate isomerase (PAI) in plants, resulting in resistance to sulfamethoxazole and 6-methylanthranilate, respectively (Han and Kim, 2019).

Genome editing, particularly programmed base editing and prime editing, is of crucial importance as it can be used to introduce heritable targeted changes that give rise to transgene-free crops (Tian et al., 2018; Butt et al., 2020; Liu et al., 2020) that are genetically indistinguishable from plants developed based on classical mutagenesis approaches (Huang et al., 2016). Furthermore, base editing facilitates the substitution of multiple amino acids in specific genes, which can contribute to enhance the resistance spectrum of crops, such as resistance to multiple diseases (Zhang et al., 2019b). Moreover, in contrast to traditionally genetically modified plants, non-transgenic plants generated using CRISPR-Cas systems are exempt from regulatory approval in different countries (Lassoued et al., 2019). Similarly, given that genome-edited crops produced without the introduction of foreign DNA do not require risk evaluation (Ishii and Araki, 2017), developers can bring new crops to the market years sooner and at considerably less cost than is the case with genetically modified crops (Waltz, 2016). Consequently, by contributing to reductions in crop development time and expense, the use of genome editing has the potential to accelerate the commercialization of crops compared with mutagenesis and conventional genetic modification.

## Conclusion

At present, the viability of the entire agriculture sector is under threat on three fronts. The evolution of new pathogens, along with the development of resistance in pre-existing pathogens, are a source of significant direct losses in crop production, whereas the depletion of environmental resources (reductions in the area of cultivable arable land and water resources) is contributing to indirect reductions in crop yield. In addition, the ever-expanding human population continues to drive the demand for sufficient food supplies to meet nutritional needs. Phytopathogens are arguably the most important causal agents of plant diseases. To date, a range of techniques have been employed to manage crop diseases, including traditional and transgenic breeding. However, although these methods have primarily remained effective, they are typically laborious and time-consuming. Recent developments in genome editing technology, particularly CRISPR-Cas9-based systems, do, nevertheless, offer considerable potential for the improvement of crop plants *via* precise trait targeting. Such genome editing can be used to introduce specific targeted modifications in terms of both gain- and loss-of-function. Moreover, these technologies enable the rapid classification of new immune receptor genes (such as guided molecular evolution and Ren-Seq) and have contributed to a significant expansion in the pool of deployable genes for enhancing resistance to a range of microorganisms. In addition, it is predicted that the development of molecular stacking and targeted gene insertion will play an increasingly important role in the generation of broad-spectrum resistance to both viral and non-viral pathogens. At the current stage of technological development, CRISPR-Cas9 systems have emerged as the most efficient and suitable alternative genome editing-based solutions for the development of disease-resistant crops, which will predictably contribute to higher crop productivity with simple and effective disease management. In addition, the emergence of new base editing systems has facilitated the development of transgene-free non-genetically modified plants, which are likely to be indistinguishable from the same plants altered using transgenic or conventional crop breeding methods. To date, several disease-resistant crops have been produced using gene editing, which will undoubtedly gain greater public acceptance than that gained by conventionally genetically modified plants. Accordingly, we firmly believe that, used responsibly, genome editing in the agricultural industry stands to make significant contributions to the enhancement of crop productivity that can benefit both producers and consumers, and it goes a long way in meeting current and future increases in human nutritional requirements.

## Author Contributions

QA, HM, and LZ planned and designed this review manuscript. QA wrote this review paper. CY, MA, MI, and MS help to draw the figures. AH, SA, XW, and MR helped to improve the manuscript writing. QA, LZ, DA, and HM contributed to the critically revising of the manuscript. All the authors have reviewed, edited, and approved the manuscript before submission.

## Funding

This work was supported by the China Postdoctoral Science Foundation (No: 2014M561669). The high-talent introduction and continuous training fund supported by Zhejiang Academy of Agricultural Sciences (No: 10300000021LL05).

## Conflict of Interest

The authors declare that the research was conducted in the absence of any commercial or financial relationships that could be construed as a potential conflict of interest.

## Publisher’s Note

All claims expressed in this article are solely those of the authors and do not necessarily represent those of their affiliated organizations, or those of the publisher, the editors and the reviewers. Any product that may be evaluated in this article, or claim that may be made by its manufacturer, is not guaranteed or endorsed by the publisher.
